# Are neuroaesthetic principles applied in art therapy protocols for neurorehabilitation? A systematic mini-review

**DOI:** 10.3389/fpsyg.2023.1158304

**Published:** 2023-06-12

**Authors:** Amelia Oliva, Marco Iosa, Gabriella Antonucci, Daniela De Bartolo

**Affiliations:** ^1^Department of Psychology, Sapienza University of Rome, Rome, Italy; ^2^Smart Lab, Scientific Institute for Research, Hospitalization and Health Care (IRCCS) Santa Lucia Foundation of Rome, Rome, Italy

**Keywords:** neuroaesthetics, neurorehabilitation, technology, virtual reality, art therapy

## Abstract

Art is an instrument created by humans as an alternative way of expression. For this reason, it has found its use in clinical contexts to improve mood, increase participation in therapy, or improve communication for patients with different pathologies. In this systematic mini-review, the Preferred Reporting Item for Systematic Reviews and Meta-Analysis (PRISMA) guidelines were adopted. Internet-based bibliographic searches were conducted via major electronic databases (Web of Science and PubMed). We analyzed the quantitative studies in which art figures as a neurorehabilitation treatment to identify whether standard art therapy protocols exist and whether these are based on the principles of neuroaesthetics. Our review identified 8 quantitative and 18 qualitative studies. Although art therapy has been used for more than 20 years as a clinical tool, there are no standard protocols to refer to when planning interventions. Although the effectiveness of using arts as therapy has been reported in many qualitative or feasibility studies, there is still a lack of quantitative studies in which the outcomes of art therapy are directly based on the principles of neuroaesthetics.

## 1. Introduction

Art activities can be considered complex, multimodal interventions that combine multiple components involving aesthetic engagement, imagination, sensory activation, the evocation of emotion, cognitive stimulation, and, possibly, social interaction, physical activity, and engagement with wellness promotion (Craig et al., 2008; Fancourt, 2017).

As art activities can trigger psychological, physiological, social, and behavioral responses, they could be linked with health and interactions with healthcare (Fancourt and Finn, 2019). In this framework, art therapy (AT) may be defined as the use of arts to support personal and relational treatment goals as well as community concerns, improve cognitive and sensorimotor functions, foster self-esteem and self-awareness, cultivate emotional resilience, promote insight, enhance social skills, reduce and resolve conflicts and distress, and even advance societal and ecological change (Regev and Cohen-Yatziv, 2018).

Art therapy can also be defined as a form of psychotherapy, physiotherapy, and/or speech therapy that uses art media as its primary mode of communication to enable the patients to develop their health and wellbeing by promoting their creative resources with art materials in a safe, enriched, and facilitating environment (Deshmukh et al., 2018).

However, in many cases, “art therapy” refers to specific treatments administered by a professional art therapist, despite the many different applications reported in the scientific literature for arts in health promotion. According to this latter general point of view, the use of arts for promoting health is a recognized therapeutic approach widely used at present in various clinical situations, especially related to neurological disorders, such as Alzheimer's disease (Davidson and Almeida, 2014), stroke (Gonen and Soroker, 2000; Beesley et al., 2011; Baumann et al., 2013), Parkinson's disease (Cucca et al., 2021), and psychiatric disorders (Grube, 2002; Caddy et al., 2012).

In 2019, the World Health Organization (WHO) published a scoping review titled “What is the evidence on the role of the arts in improving health and wellbeing?” (Fancourt and Finn, 2019). This review mainly divided the possible outcomes of arts into promotion/prevention and management/treatment. Furthermore, in this WHO document, art therapy was divided into contemplative protocols (such as watching visual arts or listening to music) and creative protocols (such as painting or playing an instrument, even if the result cannot exactly be defined as arts). Finally, this document of the WHO further divided the types of arts applied to health into five main broad categories. The categories are performing arts (e.g., activities in the genre of music, dance, theater, singing, and film), visual arts (e.g., crafts, design, painting, photography, sculpture, and textiles), literature (e.g., writing, reading, and attending literary festivals), culture (e.g., going to museums, galleries, art exhibitions, concerts, theater, community events, cultural festivals, and fairs), and digital arts (e.g., animations, film-making, computer graphics, and online fruition of arts) (Fancourt and Finn, 2019).

These five approaches may involve imagination, aesthetic engagement, sensory activation, cognitive stimulation, the evocation of emotion, and, in some cases, an increase in physical activity and social interaction (Fancourt and Finn, 2019). They may also encourage patients to develop a nonverbal language to help overcome emotional, cognitive, linguistic, or motor disturbances.

Given that the review of the World Health Organization covered more than 3,000 studies, it is quite surprising that the word “neuroaesthetics” is not present in this article. Neuroaesthetics is an emerging field of neuroscience, defined as the scientific study of the cognitive processes and the neural bases related to the contemplation and creation of a work of art (Zeki, 1999; Nalbantian, 2008).

Although art therapy could be a bridge between neuroaesthetics and neurorehabilitation, it appears that the protocols of arts for health do not benefit from the recent scientific findings in the field of neuroaesthetics. Among the discoveries in this neuroscientific field, the wide brain arousal including the activation of motor areas, even when an art masterpiece is simply contemplated by a static subject (Ishizu and Zeki, 2013), is an interesting one. Many different possible explanations have been provided for this elicitation of motor and premotor areas, including the possible activation of mirror neurons (Freedberg and Gallese, 2007), the possible recognition of the motor intentions of the painted people (Adolphs et al., 2000), the motor imagery related to the use of painted tools, the accessibility of the painted environments (Di Dio et al., 2016), or even the empathetic engagement with the artist that, with his/her gestures, produced (or played) the artistic masterpiece (Knoblich, 2002; Umiltà et al., 2012).

The possible relationship between neuroaesthetic and art-based protocols for rehabilitation is an important area of inquiry because there is a need to develop a roadmap to enhance and enrich art therapies with a greater understanding of neuroscience (King, 2018). The use of arts for health promotion originated in the 20th century in many parts of Europe and America simultaneously in response to the needs of clinical populations (often not being served effectively with traditional approaches to mental and physical health) based on the value of creative, symbolic communication, memory reconsolidation, and emotional regulation of arts. Although arts for health promotion has increasingly well-known and established potentialities, deeper research in the field is needed to better understand the neuropsychological mechanisms based on the intervention's impacts and patients' outcomes (King et al., 2019). Moreover, many studies in the field of neuroaesthetics are limited to laboratory settings, whereas they may suggest translational applications for health promotion (King and Parada, 2021).

This mini-review aimed to analyze the neurorehabilitation protocols exploiting the potentialities of arts and verify if they should translationally take into account the findings of studies conducted in the field of neuroaesthetics.

## 2. Materials and methods

This systematic review was conducted by searching recent peer-reviewed articles published until August 2022 using the PubMed and Scopus databases.

The scientific literature was reviewed by two independent researchers. In the primary search, the following keywords were used: “art therapy” AND “neurorehabilitation.” The objective of the review search was to identify papers that used a systematic protocol for neurorehabilitation based on art therapy. Moreover, rehabilitation outcomes should have been assessed and quantified using clinical scales or biomedical devices. The research question of the mini-review was whether or not art-based therapy was implemented on the neuroaesthetic principle. Therefore, this study aimed to verify the research question. After the removal of the duplicates, all articles were evaluated based on titles and abstracts. Papers were screened and included as valuable studies according to the following criteria: (i) they used art therapy as an experimental treatment; (ii) they were focused on the neurorehabilitation of adult clinical populations; (iii) the papers were written in English; (iv) they clearly reported quantitative outcome measurements; and (v) they had been published in a peer-reviewed journal. We excluded articles that described theoretical models, methodological approaches, algorithms, basic technical descriptions, letters to the editor, and validation of experimental devices without a clear translation to clinical practice and without quantitative measurements. Furthermore, we excluded papers with different populations, such as (i) animal studies; (ii) pediatric studies; and (iii) adult psychiatric research, or with a different format, such as (iv) conference proceedings or reviews and (v) papers that were not fully available. A secondary adjunctive search was made using the following keywords: “neuro-aesthetic^*^” AND (“neurorehabilitation” OR “rehabilitation” OR “therapy”). Papers were also screened from previous systematic reviews on this topic and by reading the references of selected papers. For each paper, the type of study, number of patients, therapy duration, main results, and findings have been recorded.

The collected data were summarized as mean ± standard deviation or percentage and were also reporting the statistical significance level of within- or between-group comparisons. Furthermore, a risk-of-bias analysis was conducted on the collected papers using the Risk-of-bias VISualization software (Robvis) for a systematic review (McGuinness and Higgins, 2021).

## 3. Results

**Figure 1:**
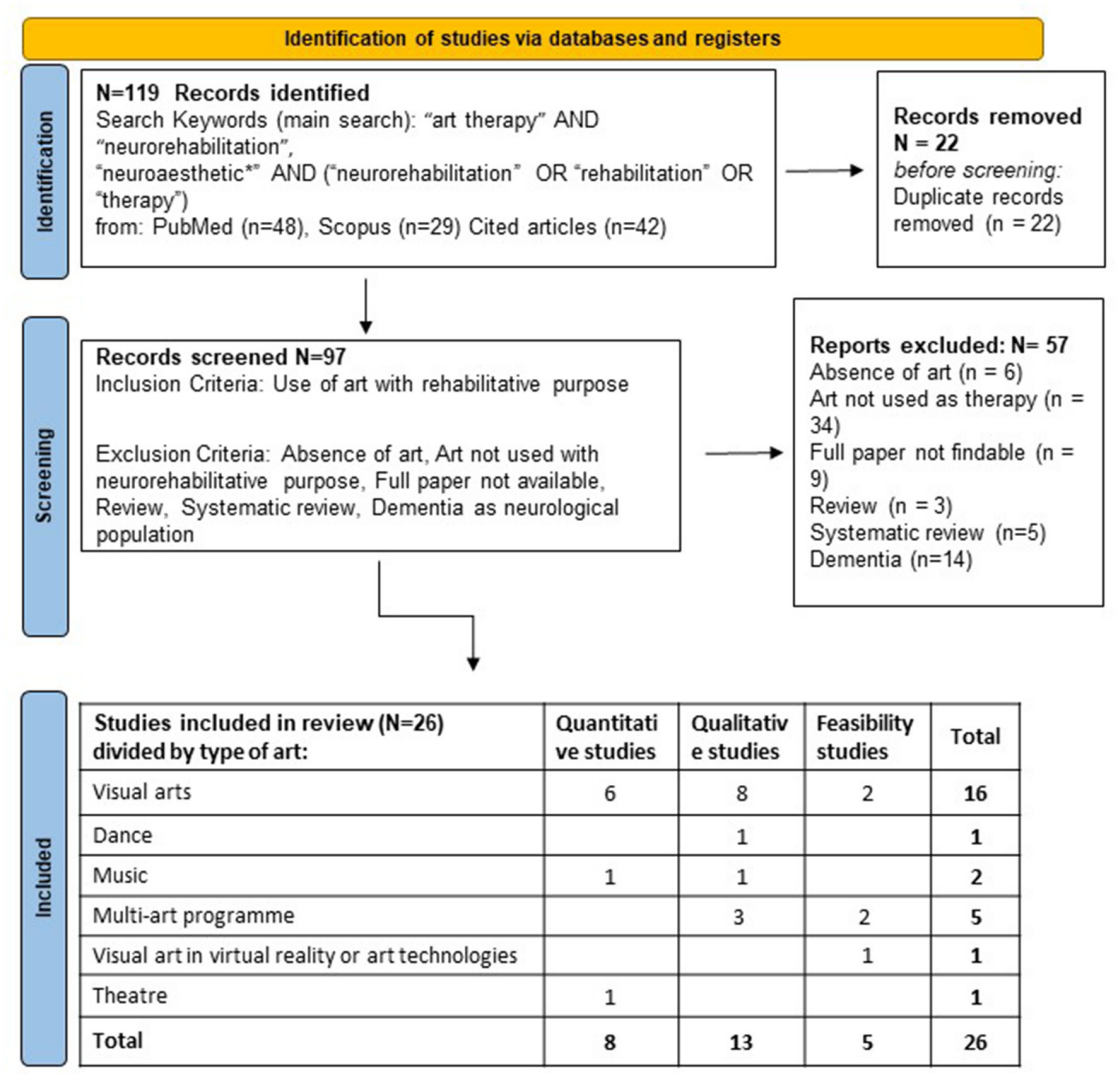
Flowchart of the mini-review described according to the PRISMA statement 2020.

**Figure 2:**
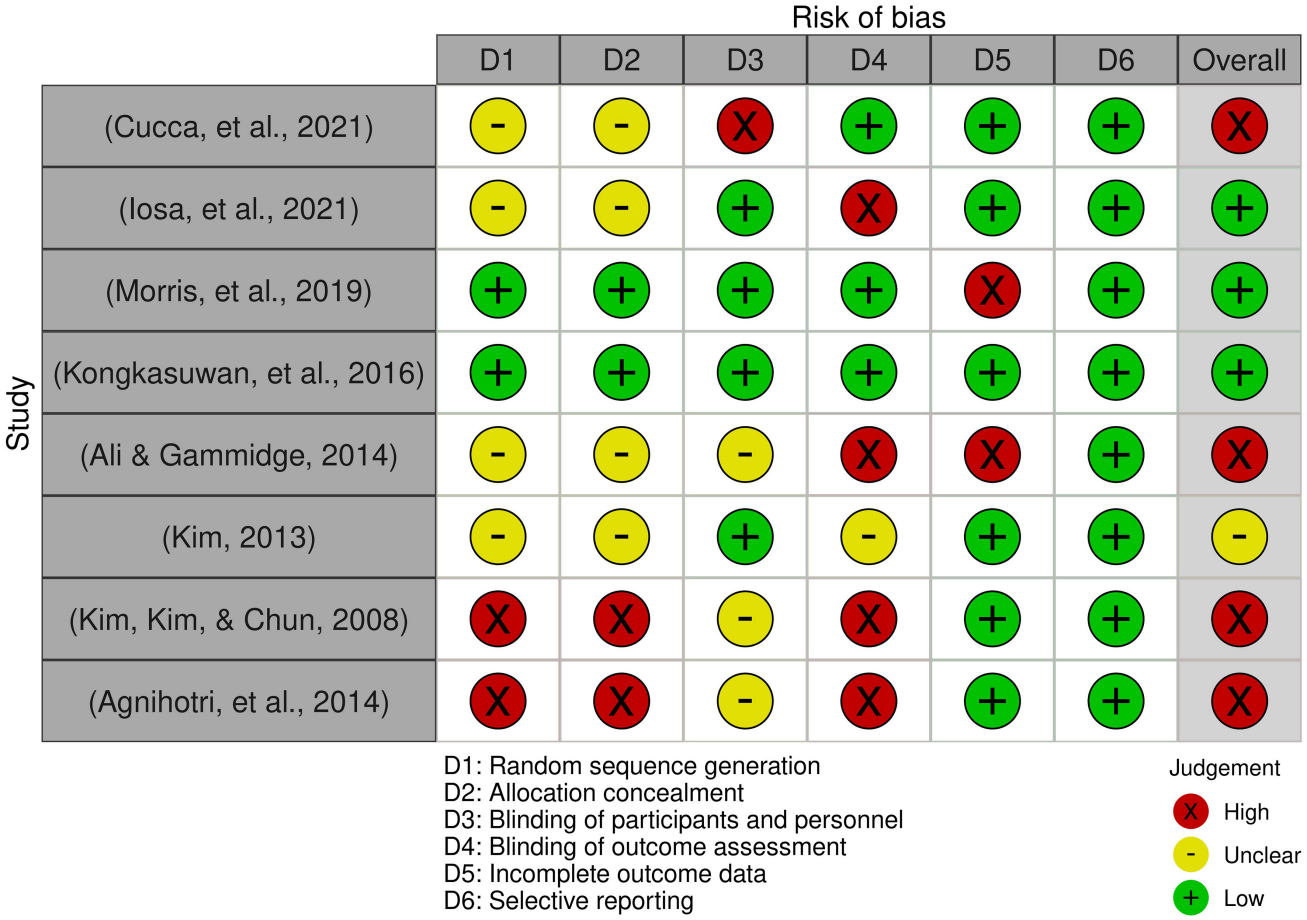
Risk-of-bias analysis for quantitative studies. This analysis was performed using Robvis (Risk-of-bias VISualization software for the R package) for systematic review (McGuinness and Higgins, 2021).

As shown in Figure 1, 119 papers have been identified. After duplication removal, 97 papers were screened, and, according to the criteria described above, 26 papers were finally selected. The results of the present search strategy are reported in Figure 1. Papers were classified by the type of arts used for rehabilitation: visual arts, music, arts in virtual reality, or therapy programs based on the combined use of various arts (visual arts, music, theater, dance, writing, etc.). Among the 26 studies, we found 8 quantitative studies (reported in Table 1), 13 qualitative studies (Carmi and Mashiah, 1996; Pachalska et al., 2008; Michaels, 2010; Beesley et al., 2011; Symons et al., 2011; Baumann et al., 2013; Vija and Lusebrink, 2014; Demers and McKinley, 2015; Morris et al., 2016; Sit et al., 2017; Smith et al., 2017; Vaudreuil et al., 2019), and 5 feasibility studies (Worthen-Chaudharia et al., 2013; Morris et al., 2014; Ellis-Hill et al., 2015; Cucca et al., 2018; Chan et al., 2021) (Supplementary material). We performed a risk of bias analysis on the quantitative studies shown in Figure 2. The colors of the traffic light in Figure 2 indicate that half of them had a high risk of bias, i.e., for an unclear study, while only 3 (Kongkasuwan et al., 2016; Morris et al., 2019; Iosa et al., 2021) out of 8 studies had a low risk of bias. Quantitative studies used art therapy in patients with Parkinson's disease, stroke, or acquired brain injury. By analyzing the quantitative, qualitative, and feasibility studies, we found that visual arts are the most commonly used (15 studies out of 26, 58%), also in their digital version (*n* = 2, 8%), followed by multi-modal art protocols (*n* = 5, 20%), music (*n* = 2, 8%), and sporadic experiences related to dance (*n* = 1, 4%) and theater (*n* = 1, 4%).

**Table 1:** Detailed information of the included quantitative studies.

**References**	**Type of art therapy**	**Type of study**	**Sample size**	**Therapy duration**	**Results**	**Findings**
Cucca et al. (2021)	Visual art (Clay manipulation, painting on canvas, collage, drawing, and murals)	Open-label, prospective, exploratory trial No CG	*N* = 18 PD patients	2 sessions per week for 2 weeks	Number of saccades _w_: *(19.9 ± 1.0, t(9) = 4.8; p = 6.2e-08);* Saccadic path lengths _w_: *(1,621 ± 113; t_(9)_ = 0.9; p = 3.1e-09)* Horizontal fixation variation _w_: *(6.8 ± 0.25; t_(9)_ = 2.5; p = 0.017)* Navon test _w_: *t_(10)_ = 1,601; p = 0.138* RCFT _w_*: t_(13)_ = 2.0295, p = 0.0634* UPDRS-III _w_: *t_(13)_ = −6.16; p = 0.0063* UPDRS totale _w_: *t_(13)_ = −6.93; p = 0.0368* Increases of FC in left paracentral lobule within V1 (*p* < 0.001); left middle temporal gyrus in V2 (*p* < 0.001)	Improvement of visual-cognitive skills, general motor function, and functional reorganization of visual networks
Iosa et al. (2021)	Visual digital art (painting on virtual canvas)	RCT CG: interaction with non-artistic masterpieces	*N* = 20 controls (Exp. 1) and 4 stroke patients (Exp. 2)	2 sessions on the same day (Exp. 1) 4 sessions in 8 days (Exp. 2)	Exp. 1 Physical demand *_*B*_*(22.1 ± 21.7% vs. 27.1 ± 18.9% *p = 0.049*) Exp.2 USEQ *mean score = 4.75* NASA TLX: *mental demand* = 9%, *physical demand* = 6%, *temporal demand* = 4%, *self-assessed performance* = 93%, *effort* = 5%, *frustration* = 1%. Pittsburgh Rehabilitation Participation Scale: mean score *ranged from 5.25 to 6 among the 4 sessions*	Reduction of perceived fatigue and kinematic errors with artistic stimuli.
Morris et al. (2019)	Visual arts (drawing, collage, printing)	RCFT CG: usual care	*N* = 81 stroke patients	2 sessions per week for 4 weeks	T1-T2: Positive affect *_*B*_(5.4 ± 9.2 vs. 1.7 ± 9.9)* Negative affect *_*B*_ (3.2 ± 10.8 vs. 4.5 ± 9.4)* Self-efficacy for art *_*B*_ (5.4 ± 9.2 vs. 1.79 ± 9.9)* T2-T3: Positive affect *_*B*_(4.3 ± 7.5 vs. 2.8 ± 10.1)* Negative affect *_*B*_ (3.3 ± 11.0 vs. 5.2 ± 9.8)* Self-efficacy for art *_*B*_(2.1 ± 4.1 vs. 0.4 ± 3.9)*	Increase in self-efficacy and emotional wellbeing
Kongkasuwan et al. (2016)	Music (meditation with music, group singing)	RCT CG: conventional physical therapy	*N* = 118 stroke patients	2 sessions per week for 4 weeks	Functional score *_*B*_ (1.2, 95% CI 0.1, 2.3, p = 0.043)*, Depression *_*B*_ (−4.5, 95% CI −6.5, 2.5, p < 0.001)*, QoL *_*B*_ (8.9, 95% CI 3.8, 13.8, p < 0.001)*	Decrease depression and increase physical functions and quality of life
Ali and Gammidge (2014)	Visual arts (drawing and painting, camera and iPad)	Pilot study CG: usual care	*N* = 27 stroke patients	2 sessions per week for 6 weeks	TOMs *_*B*_*: *from 9 to 10.5* HAD anxiety *_*B*_*: *from 8 to 6* HAD depression *_*B*:_ from 10 to 4*	Art therapy helps patients to explore the sequel of stroke
Kim and Kang (2013)	Visual art (colors therapy)	RCT CG: comprehensive rehabilitation	*N* = 28 stroke patients and their 28 caregivers	1 session a week for 13 weeks	Meaning in life *_*B*_ P 40.3 ± 3.6 p < 0.05 C 37.4 ± 2.1 p < 0.05* Life value selection *_*B*_ P 24.6 ± 4.9 p < 0.05 C 24.6 ± 1.6 p < 0.05* Aim of life *_*B*_ P 45.6 ± 5.9 p < 0.05 C42.7 ± 2.2 p < 0.05*	Color therapy improves the purpose in the life of post-stroke patients and caregivers
Kim et al. (2008)	Visual art (drawing)	Case report	*N* = 1 stroke patient	Twice a week for 10 weeks	K-MMSE: from 0 to 6 K-WAIS: from 40 to 59 K-WAB: from 16.8 to 17.0 MVPT: from unmeasurable to measurable Fugl-Meyer: from 6 to 32 FIM: from 41 to 57	Improvement in visual perception, cognition, motor activity, and function
Agnihotri et al. (2014)	Theater (voice work, character development, script analysis, writing skills)	Case study	*N* = 4 youth with ABI *N* = 1 control	Daily for 4 h over a period of 4 weeks	COPM, GAS, Social Networks Inventory CASP, RSES, Emotion Discrimination Task PPIC	Improvement of social skills and participation

RCT, randomized controlled trial; RCFT, randomized controlled feasibility trial; PD, Parkinson's disease; ABI, acquired brain injury; RCFT, Rey-Osterrieth Complex Figure test; UPDRS, Unified Parkinson's Disease Rating Scale; FC, functional connectivity; V1, V2, primary and secondary visual brain networks; USEQ, user satisfaction evaluation questionnaire; NASA TLX, Nasa task load index; QoL, quality of life; TOM, therapy outcome measures; HADS, Hospital Anxiety and Depression Scale; K-MMSE, the Korean version of Mini-Mental Status Examination; K-WAIS, the Korean version of Wechsler Adult Intelligence Scale; K-WAB, the Korean version of Western Aphasia Battery; MVPT, Motor-Free Visual Perception Test; FIM, Functional Independence Measure; COPM, Canadian Occupational Performance Measure; GAS, Goal Attainment Scaling; CASP, Child and Adolescent Scale of Participation; RSES, Rosenberg Self-Esteem Scale; PPIC, Profile of Pragmatic Impairments in Communication; CG, control group; _w_, within group study; _B_, between group study.

In quantitative studies, the outcomes were evaluated using clinical scales, psychological and behavioral tests, instrumented movement analysis, neuroimaging, and eye-tracking analyses. Significant improvements were obtained with art therapy in terms of functional connectivity (Cucca et al., 2021), reduction of anxiety and depression (Ali and Gammidge, 2014; Kongkasuwan et al., 2016), and reduction of fatigue during therapy (Iosa et al., 2021) but not in terms of the activities of daily living (Cucca et al., 2021) and increment in participation in the therapeutic intervention (Agnihotri et al., 2014; Morris et al., 2019). Specific studies also reported improvements in visual perception (Kim et al., 2008), level of purpose in life (Kim and Kang, 2013), and self-assessed concentration, emotion, self-confidence, motivation, and satisfaction with respect to art therapy (Kongkasuwan et al., 2016).

Findings of qualitative and feasibility studies have been reported in a table of Supplementary material together with the details of the risk of bias analysis, which revealed that only 5 out of the 18 studies had a low risk of bias (28%).

## 4. Discussion

The main result of our review is that although the studies included in this review used a quantitative approach, it appears that they did not begin with the principles of neuroaesthetics to define systematic protocols of art therapy for neurorehabilitation programs. In addition, there were twice the number of qualitative and feasibility studies than quantitative ones. These two aspects could be strictly intertwined. Art-therapy protocols are often neither based on rigorous protocols based on scientific literature nor included quantitatively and objectively measured outcomes.

According to the analyzed studies, the patient can express internal conflicts, emotions, and his own psychological state through the work of art (Ali and Gammidge, 2014). In other words, art therapy can also be used as a form of group therapy, promoting interaction and communication between patients (Goodill, 2010) and reducing anxiety, depression, and isolation (Eum and Yim, 2015). Creativity is associated with flexible or divergent thinking (Torrance, 1995). It can also help caregivers gain different perspectives on the challenges they face and find new and flexible ways to adapt to their role as caregivers (Houston, 2020).

However, most of the analyzed studies are characterized by small groups of participants or the absence of control groups, which implies poor statistical rigor or a lack of an indicator of the obtained result to support the reported findings quantitatively. In the absence of psychological measures on motivation, participation, and many other cognitive aspects that have been reported as positively influenced by the art experience (Kongkasuwan et al., 2016), the findings of qualitative studies risk remaining positive anecdotic experiences with poor generalizability of the scientific outcomes (Slayton et al., 2010).

The absence of protocols based on solid principles extracted by neuroaesthetic and neuroscientific studies reporting quantifiable outcomes, even for quantitative studies, may be the basis of some skepticism concerning the efficacy of art therapy in neurorehabilitation. In contrast, neuroaesthetics is mainly investigated in studies on general psychology and on healthy subjects, with a poor interest in its application to art therapy in the broadest sense with translational applications to rehabilitation practice (Babiloni et al., 2014; Di Dio et al., 2016; De Bartolo et al., 2022).

A potential bias of our review is the search for art therapy as a generic term, despite some studies that could refer only to specific types of arts, for example, music therapy or dance therapy. However, we aimed to identify studies with a quantitative approach that may have considered neuroaesthetic principles to develop protocols for art therapy. Another aspect to take into account is that our review was mainly focused on neurorehabilitation due to its direct link to neuroscience.

The main finding of our review is that neurorehabilitation protocols exploit the potentialities of arts in increasing the cognitive and emotional engagement of patients that need neurorehabilitation badly while taking into account neuroaesthetic principles. According to the analyzed literature, art therapy is currently widely used in the care of those patients whose impairment is high, such as patients with severe neurological or psychiatric cognitive impairment, in which a direct approach, such as that required by neurorehabilitation, is not possible. In line with these considerations, further evidence is needed; however, we can argue that the use of art therapy could also help patients who have suffered from motor impairment to manage their mood and improve their participation in neuromotor therapy (Iosa et al., 2021).

In conclusion, this mini-review highlighted a gap between the many different protocols using arts for promoting health in neurorehabilitation and the rigorous findings of the study of neuroaesthetics, which are often lacking in translational applications.

As demonstrated by the risk of bias analysis, quantitative studies also have some limitations. Random sequence generation is not present in most of the included studies (Kim et al., 2008; Kim and Kang, 2013; Agnihotri et al., 2014; Ali and Gammidge, 2014; Cucca et al., 2021; Iosa et al., 2021). This implies that learning from having to repeat the same task may have affected the patient's performance. Future studies should take this aspect into account and provide randomization of the stimuli to keep disturbing variables under control. Similarly, in these studies, randomization between the experimental and control groups was not foreseen (or was not declared). This may have affected the participants' performance and their motivation to participate in an experimental or control group. This latter is not always present (Kim et al., 2008; Agnihotri et al., 2014; Cucca et al., 2021) and usually follows normal conventional therapy (Kim and Kang, 2013; Ali and Gammidge, 2014; Kongkasuwan et al., 2016; Morris et al., 2019), so there is no direct comparison between art therapy vs. non-art stimuli, as reported by Iosa et al. (2021).

Given that arts appear to have the potentialities to increase participation, motivation, and confidence during therapy, a more solid approach, based on neuroaesthetic principles and an objective assessment of the outcomes, could be helpful to empower these results and favor the use of arts for improving patients' health and wellness.

## Author contributions

MI conceptualized the review. AO and DDB conducted the literature researches and wrote the first draft of this manuscript. GA and MI revised it providing conceptual contributions.
