# Supplementary material for: Are neuroaesthetic principles applied in art therapy protocols for neurorehabilitation? A systematic mini-review
Source: Front Psychol. 2023 Jun 12;14:1158304. doi: 10.3389/fpsyg.2023.1158304 (PMC10291050; doi:10.3389/fpsyg.2023.1158304)

***Supplementary Material***

*Table S1. Detailed information of the included qualitative and feasibility studies. RCFS, Randomised Controlled Feasibility Study. PD, Parkinson Disease. CG, Control Group*

| **Study** | **Therapy intervention** | **Type of study** | **Number of patients** | **Therapy duration** | **Results** |
| --- | --- | --- | --- | --- | --- |
| Chan, et al., 2021. | Multi-arts program (Music, dance, visual arts, theatre, writing) | RCFS  CG: usual treatment | N= 154 stroke patients | 1 session per week for 8 weeks | No results |
| Cucca, et al., 2018. | Visual arts (Oils, pastels, clay, ornamental fabrics, watercolor, paint) | Open-label, prospective, exploratory feasibility study  CG: no art assessment | N = 20 PD and 20 controls | 2 sessions per week for 10 weeks | No results |
| Sit, et al., 2017. | Visual arts (Clay making, coloring, photos decoration, drawing) | Exploratory, qualitative design  No CG | N= 24 stroke patients | 1 session a week for 7 weeks | Improvements of the sense of enjoyment and self-expression. |
| Morris, et al., 2016. | Visual arts (Drawing and painting, printing, textiles, 3D modelling) | Exploratory, qualitative design  No CG | N= 11 stroke patients | 40 min one-to-one and 90 min group sessions per week (up to 8 sessions) | Improvement of stroke survivors’ mood, confidence and self-esteem |
| Ellis-Hill, et al., 2015. | Multi-arts program (Colours, drawing materials, clay, textiles, books, poems, pictures, music, films) | RCFS  No CG | N= 64 stroke patients | 10 two-hour group sessions over the course of 14 weeks | No results |
| Morris, et al., 2014. | Visual arts (Drawing, collage, printing, painting and mixed-media technique) | RCFS  CG: usual care | N= 80 stroke patients | up to 8 sessions (4 within a group and 4 one-to-one) in 12 weeks | No results |
| Baumann, Peck, & Collins, 2013. | Multi-arts program (Visual images, music, poems, building materials) | Qualitative study  No CG | N= 16 stroke patients | 1 session per week for 5 weeks | Engagement in a pleasant and purposeful occupation and reconnection with valued aspects of oneself |
| Beesley et al., 2011. | Multi-arts program (Painting on canvas, charcoal, wax resist and drawing) | Qualitative study  No CG | N= 16 stroke patients | 2h per week (tot 8 weeks) | Improvement of confidence, self-efficacy, quality of life and community participation |
| Pachalska, Grochmal-Bach, & Wilk, 2008. | Visual art (Personalized programme based on patient’s paintings) | Case report | N= 1 stroke patient (the artist Krystyna Habura) | 5-stage program lasting 8 weeks | Krystyna Habura was able to overcome the loss of artistic vision in her brain |
| Vaudreuil, Avila, & Pasquina, 2019. | Music | Case report | N= 1 (Army CPT Luis Avila) | two to three times per week per 60-min for 2 years | Improvement of functional use of the upper limbs, social integration, quality of life and motivation |
| Worthen-Chaudharia, Whalenb, Swendala, Bockbradera, & Haserodtc, 2013. | Interactive arts technologies (Computer graphic art) | Feasibility study | N = 21 | 60 min per session  (1 to 7 session) | Direct observation:  Improvement of ability to solve movement problems autonomously. |
| Carmi and Mashiah, 2013. | Visual art (Painting) | Case report | N= 1 stroke patient | Many hours a day during his hospitalization | Improvement of the recognition and organization of the internal conflicts |
| Vija B. Lusebrink, 2014. | Visual art (Collage, clay) | Case report | N= 1 chronic schizophrenia patient  N= 1 acute schizophrenia patient | No information | Art therapy offers the possibility to deal with basic sensory building blocks in the processing of information and emotions |
| Michaels, 2010. | Visual art (Drawing) | Case report | N= 1 stroke patient | 2 weeks | Art therapy offers a potential ‘space for linking’ shattered and meaningless experience, by mediating between mind and body, self and other, past and present. |
| Smith, Wright, Lakhani, & Zeeman, 2017.] | Visual art (Drawing with pastels) | Pilot study  No CG | N= 3 adults with ABI | 2 sessions | Art processes may give voice to people with limited capacity to verbalize |
| Symons, Clark, Williams, Hansen, & Orpin, 2011.[42] | Visual arts (Acrylic and watercolor painting) | Qualitative study  No CG | N= 9 patients with neurological conditions | one session a week for 6 months | Art increases enjoyment and self-confidence and facilitates planning for engagement in future activities |
| Demers & McKinley, 2015. | Dance (Combination of jazz dance and merengue) | Qualitative study  No CG | N= 9 stroke patients | biweekly 45-min for 4 weeks | Participants perceived dance as an enjoyable social and physical activity |

Figure S1. Risk of Bias Analysis for qualitative and feasibility studies. This analysis was performed using Robvis (Risk‐of‐bias Visualization software for the R package) for systematic review (McGuinness & Higgins, 2021).


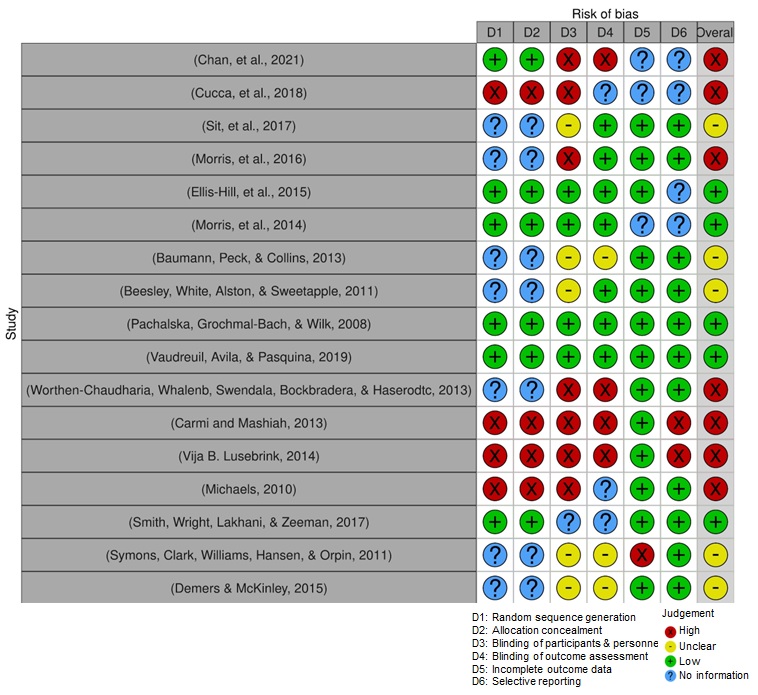

Supplement: Supplementary file 1 [file Data_Sheet_1.docx]
